# Supplementary material for: MicroRNA Profile of Human Small Intestinal Tumors Compared to Colorectal Tumors
Source: J Clin Med. 2022 May 6;11(9):2604. doi: 10.3390/jcm11092604 (PMC9103422; doi:10.3390/jcm11092604)
Supplement: Supplementary file 1 [file jcm-11-02604-s001.zip › jcm-1697251-supplementary.pdf]

Journal of
Clinical Medicine

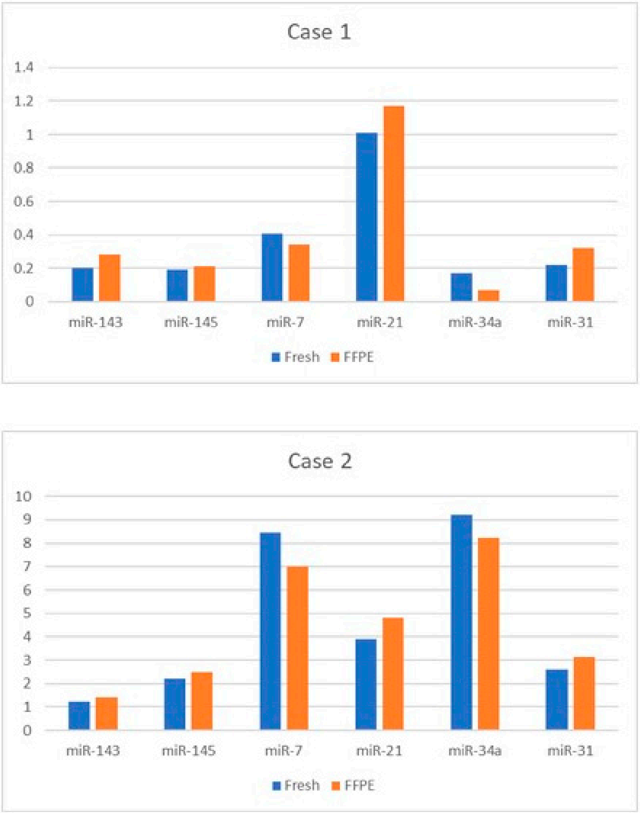

Figure S1. miRNA profiles between FFPE and fresh samples in the same sample of small intestinal tumor.
